# P-666. Human Challenge Models Using Current Clinical Strains of Influenza, RSV, hMPV, and SARS-CoV-2 Omicron

**DOI:** 10.1093/ofid/ofaf695.879

**Published:** 2026-01-11

**Authors:** Nicolas Noulin, Mariya Kalinova, Alexander J Mann, Brandon Londt, Andrew P Catchpole

**Affiliations:** hVivo Services LTD, London, England, United Kingdom; hVivo Services Ltd., London, England, United Kingdom; hVIVO, London, England, United Kingdom; hVivo, London, England, United Kingdom; hVivo Services Ltd., London, England, United Kingdom

## Abstract

**Background:**

Human challenge models (HCMs) offer a controlled, accelerated approach to evaluating vaccines and antivirals for respiratory pathogens. We report the development of a comprehensive HCM platform using contemporary clinical strains of influenza A (H1N1, H3N2), influenza B, SARS-CoV-2 Omicron (BA.5), respiratory syncytial virus A and B (RSV-A, RSV-B), and human metapneumovirus (hMPV), all of which remain major contributors to global respiratory disease burden.

**Methods:**

Clinical isolates representing currently circulating strains were cultured under GMP conditions following extensive in vitro characterization. Challenge studies in healthy adults were conducted for each pathogen to assess safety, determine optimal dosing, and characterize infection kinetics. Volunteers were intranasally inoculated and quarantined for 8–15 days, with continuous monitoring via symptom diaries, virologic assessments (qPCR and culture), bloodwork (CBC, CRP), ECGs, spirometry, and serial collection of serum and PBMCs for immunological profiling.

**Results:**

Each pathogen successfully induced infection and measurable clinical symptoms in a relevant proportion of participants, with distinct temporal and symptom profiles. All studies were completed without serious adverse events. Viral shedding closely mirrored symptom onset and resolution. Immune profiling demonstrated variable baseline immunity across pathogens, with observable boosting of neutralizing antibodies and T-cell responses post-challenge. Omicron BA.5 induced shorter, milder illness than historic strains.

**Conclusion:**

We successfully established a multi-pathogen Human Challenge Model platform leveraging recent, clinically relevant respiratory virus strains. This platform enables head-to-head comparisons of host responses and supports the rapid evaluation of vaccines, monoclonal antibodies, and antivirals targeting respiratory viruses.

**Disclosures:**

Alexander J. Mann, BSc (Hons), hVivo Ltd.: Employee of hVivo LTD Brandon Londt, PhD, hVivo Ltd: Employee of hVivo LTD Andrew P. Catchpole, PhD, hVivo Ltd: Employee of hVivo LTD

